# Retraction: SHCBP1 promotes papillary thyroid carcinoma carcinogenesis and progression through promoting formation of integrin and collagen and maintaining cell stemness

**DOI:** 10.3389/fendo.2024.1379880

**Published:** 2024-02-13

**Authors:** Frontiers Editorial Office

The journal retracts the 26 February 2021 article cited above.

Following publication, concerns were raised regarding the integrity of the images in the published figures. The authors failed to provide a satisfactory explanation during the investigation, which was conducted in accordance with Frontiers’ policies.

This retraction was approved by the Chief Editors of Frontiers in Endocrinology and the Chief Executive Editor of Frontiers. The authors have not responded to correspondence regarding this retraction.

